# Emergence of an Extensive Drug Resistant Pseudomonas aeruginosa Strain of Chicken Origin Carrying *bla*_IMP-45_, *tet*(X6), and *tmexCD3*-*toprJ3* on an Inc_pRBL16_ Plasmid

**DOI:** 10.1128/spectrum.02283-22

**Published:** 2022-10-27

**Authors:** Ning Dong, Congcong Liu, Yanyan Hu, Jiayue Lu, Yu Zeng, Gongxiang Chen, Sheng Chen, Rong Zhang

**Affiliations:** a Department of Medical Microbiology, School of Biology and Basic Medical Science, Medical College of Soochow University, Suzhou, China; b Suzhou Key Laboratory of Pathogen Bioscience and Anti-infective Medicine, Soochow University, Suzhou, China; c Department of Infectious Diseases and Public Health, Jockey Club College of Veterinary Medicine and Life Sciences, City University of Hong Kong, Kowloon, Hong Kong; d Department of Clinical Laboratory, Second Affiliated Hospital of Zhejiang University, School of Medicine, Zhejiang, Hangzhou, China; Instituto de Higiene

**Keywords:** *Pseudomonas aeruginosa*, *bla*_IMP-45_, extensive drug resistance, *tet*(X6), *tmexCD3-toprJ3*

## Abstract

This study reports an extensively drug resistant Pseudomonas aeruginosa strain PA166-2 which was of chicken origin and carrying *bla*_IMP-45_, *tet*(X6) and *tmexCD3*-*toprJ3* on a single plasmid. The strain was characterized by antimicrobial susceptibility testing, resistance gene screening, conjugation assay, whole-genome sequencing, and bioinformatics analysis. Strain PA166-2 was resistant to tigecycline and carbapenems. It belonged to ST313 and carried a plasmid pPA166-2-MDR, which belongs to the incompatibility group Inc_pRBL16_. pPA166-2-MDR harbored a 78 Kb multidrug resistance (MDR) region carrying an array of antimicrobial resistance genes, including *bla*_IMP-45_, *tet*(X6), and *tmexCD3*-*toprJ3*. The gene *bla*_IMP-45_ was inserted into the backbone of plasmid pPA166-2-MDR within a class 1 integron, In*786*. *tmexCD3*-*toprJ3* in plasmid pPA166-2-MDR was inserted in *umuC*, constituting the genetic context of IS*Cfr1*-*tnfxB3*-*tmexC3*-*tmexD3*-*toprJ3*-△*umuC*. The genetic context of *tet*(X6) in this plasmid was identical to that of other reported plasmid-borne *tet*(X) variants, namely, *tet*(X6)-*abh*-*guaA*-IS*Vsa3*. To the best of our knowledge, this is the first report of the cooccurrence of *bla*_IMP-45_, *tet*(X6), and *tmexCD3*-*toprJ3* in one plasmid in Pseudomonas sp. The emergence of plasmid-mediated tigecycline resistance genes *tmexCD3*-*toprJ3* and *tet*(X6), as well as carbapenemase genes from chickens expanded the global transmission of vital resistance genes. Findings from us and from others indicate that plasmids of the incompatibility group Inc_pRBL16_ may serve as a reservoir for carbapenem and tigecycline resistance determinants.

**IMPORTANCE** Pseudomonas aeruginosa is an opportunistic pathogen that causes infections that are difficult to treat. This study reported, for the first time, the occurrence of last-resort antibiotic resistance determinants *bla*_IMP-45_, *tet*(X6), and *tmexCD3*-*toprJ3* on a single plasmid in P. aeruginosa from chickens. The P. aeruginosa strain belonged to ST313 and was resistant to last-line antibiotics, namely, carbapenems and tigecycline. The plasmid carrying the last-line resistance genes belonged to the incompatibility group Inc_pRBL16_, which was reported to contain different profiles of accessory modules and thus carried diverse collections of resistance genes. The emergence of plasmid-mediated tigecycline resistance genes *tmexCD3*-*toprJ3* and *tet*(X6), as well as carbapenemase genes, from chickens expanded the global transmission of vital resistance genes. The results in this study highlighted that Inc_pRBL16_ plasmids may serve as a reservoir for the dissemination of resistance genes. Control measures should be implemented to prevent the further dissemination of such strains.

## OBSERVATION

Pseudomonas aeruginosa is a leading cause of morbidity and mortality in cystic fibrosis patients and immunocompromised individuals (1). The treatment of P. aeruginosa infections has become a significant challenge due to its remarkable capacity to resist many of the currently available antibiotics (2). P. aeruginosa exploits intrinsic, acquired, and adaptive resistance mechanisms to counter antibiotic attacks (3). Efflux pumps belonging to the plasmid-mediated resistance–nodulation–division (RND) family play a prominent role in the multidrug resistance of P. aeruginosa. Recently, a novel RND-type efflux pump gene cluster, *tmexCD1*-*toprJ1*, and its homologs, *tmexCD2*-*toprJ2* and *tmexCD3*-*toprJ3*, were reported to confer resistance to different classes of antibiotics, including the last-line antibiotic, tigecycline (4–7). The *tmexCD*-*toprJ* gene clusters were speculated to have originated from the chromosome of a Pseudomonas species and disseminated among diverse bacterial species, including Pseudomonas sp., Klebsiella sp., *Aeromonas* sp., Enterobacter sp., Proteus sp., and Raoultella sp. (4–8). The coexistence of *tmexCD*-*toprJ* with other antimicrobial resistance genes, such as the colistin resistance gene *mcr*, the high-level mobile tigecycline resistance gene *tet*(X), and the carbapenemase genes *bla*_NDM_ and *bla*_KPC_ in single isolates, has been reported, particularly in Klebsiella sp. (5, 9, 10). The spread of mobile elements cobearing different last-line antimicrobial resistance determinants seriously compromises the effectiveness of clinical therapy. In this study, we report an XDR P. aeruginosa strain that co-harbors *bla*_IMP-45_, *tet*(X6), and *tmexCD3*-*toprJ3* on an Inc_pRBL16_ plasmid of chicken origin. Heightened efforts are needed to control the dissemination of such strains.

P. aeruginosa strain PA166-2 was isolated from the cloaca swab of a chicken in a poultry farm in Shanxi, China in 2019. Antimicrobial susceptibility testing was conducted via the broth dilution method, and the results suggested that PA166-2 was resistant to tetracyclines (doxycycline and minocycline), a glycylcycline (tigecycline), carbapenems (meropenem and imipenem), some β-lactams (ceftazidime, cefepime, piperacillin-tazobactam, cefoperazone/sulbactam, ceftazidime-avibactam), ciprofloxacin, and an aminoglycoside (amikacin). The strain also exhibited intermediate resistance to colistin. However, the strain remained susceptible to aztreonam. The antimicrobial resistance profiles and mechanisms of resistance of P. aeruginosa PA166-2 are shown in Table 1. According to the nonsusceptibility level of strain PA166-2, it was classified as an extensive drug resistant (XDR) strain which was resistant to at least one agent in all but two or fewer antibiotic categories. Carbapenem resistance in P. aeruginosa is frequently associated with the expression of carbapenemase genes, so genes, including *bla*_IMP_, *bla*_NDM_, *bla*_VIM_, *bla*_KPC_, and *bla*_OXA_, were screened via polymerase chain reaction (PCR) and Sanger sequencing, using primers described previously (11). A *bla*_IMP_ gene was detected positive. Meanwhile, strain PA166-2 was positive for the RND-type efflux pump gene cluster *tmexCD*-*toprJ* and for the *tet*(X) gene, both of which were recently reported to have conferred resistance to tigecycline (4, 12, 13). The antimicrobial resistance gene screening results were in line with the antimicrobial susceptibility testing (AST) results.

**TABLE 1:** Results of antimicrobial susceptibility tests and genetic characterization^*a*^

Antimicrobial agents	MIC (mg/L)	Interpretation	Mechanism of resistance/location of resistance gene
Aminoglycosides			
Amikacin	≥128	R	*aph(3′)-VIa*, *aph(3′)-Ic*, *aph(4)-Ia*, *armA*, *aac(3)-Iva*, *ant(3″)-Ih-aac(6′)-IId*, /plasmid; *aph(3′)-IIb*/chromosome
β-Lactams			
Imipenem	4	R	*bla*_IMP-45_/plasmid
Meropenem	32	R	*bla*_IMP-45_/plasmid
Ceftazidime	>128	R	*bla*_IMP-45_/plasmid
Cefepime	128	R	*bla*_IMP-45_ and *bla*_OXA-1_/plasmid
Piperacillin-tazobactam	128/4	R	*bla*_IMP-45_ and *bla*_OXA-1_/plasmid
Cefoperazone/sulbactam	>128/64	R	*bla*_IMP-45_/plasmid
Ceftazidime-avibactam	>64/4	R	*bla*_IMP-45_/plasmid
Aztreonam	≤4	S	-
Fluoroquinolones			
Ciprofloxacin	16	R	*qnrVC1* and *tmexCD3*-*toprJ3*/plasmid
Tetracyclines			
Doxycycline	>32	R	Intrinsic resistance; *tet*(C), *tet*(X6) and *tmexCD3*-*toprJ3*/plasmid
Minocycline	32	R	Intrinsic resistance; *tet*(C), *tet*(X6) and *tmexCD3*-*toprJ3*/plasmid
Glycylcyclines			
Tigecycline	16	R	Intrinsic resistance; *tmexCD3*-*toprJ3* and *tet*(X6)/plasmid
Polymyxins			
Colistin	1	I	-
Trimethoprim-sulfamethoxazole			
Not included in the AST panel	NA	NA	*sul1* and *dfrA22e*/plasmid
Macrolide, lincosamide and streptogramin B antibiotics			
Not included in the AST panel	NA	NA	*msr*(E) and *mph*(E)/plasmid

a R, resistant; S, susceptible; I, intermediate; NA, not applicable; -, none.

**FIG 1:**
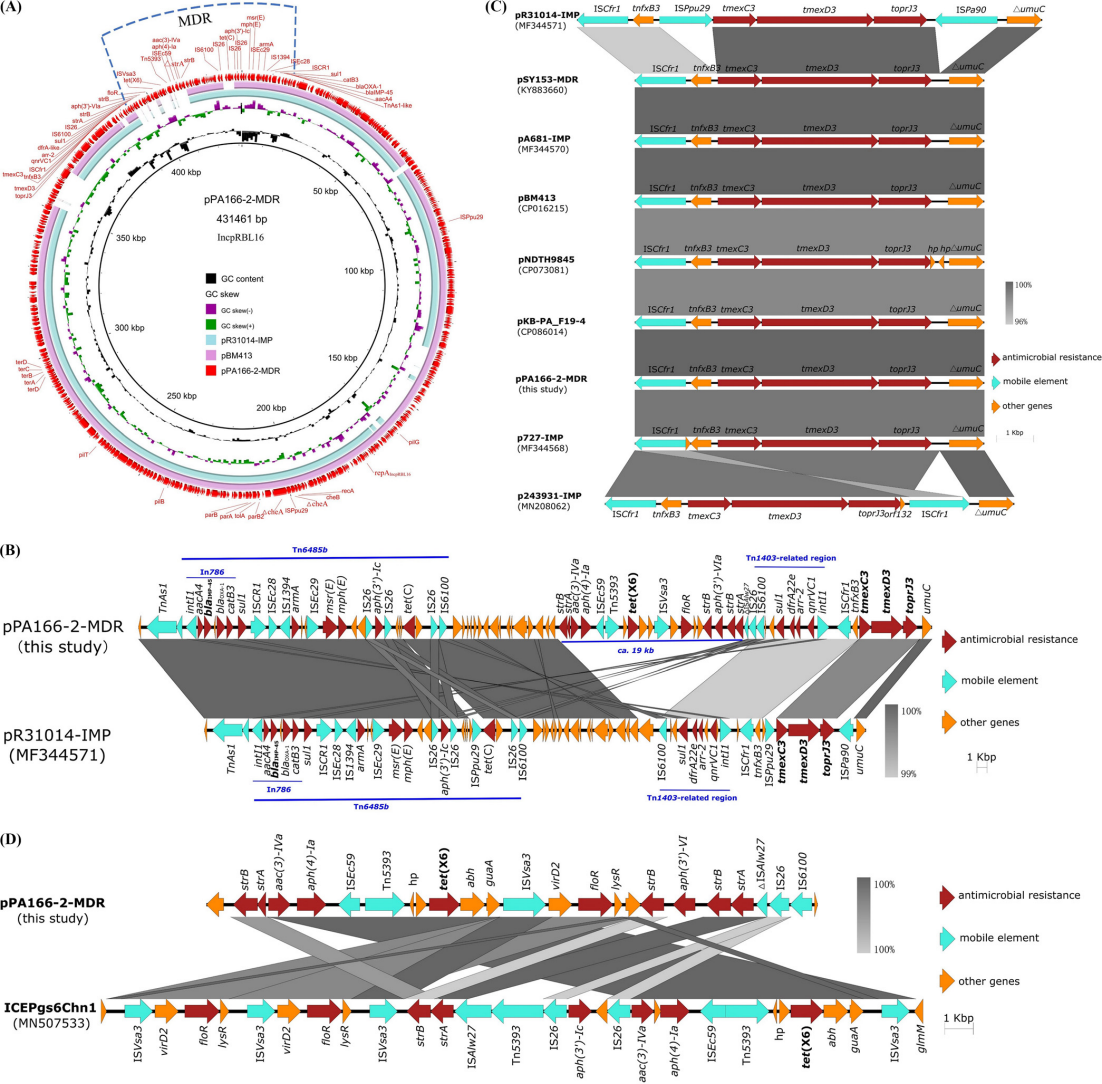
Detailed information regarding plasmid pPA166-2-MDR from Pseudomonas aeruginosa carrying an MDR region that encodes *bla*_IMP-45_, *tet*(X6) and *tmexCD3*-*toprJ3*. (A) Comparative structural analysis of pPA166-2-MDR with other similar plasmids available in the NCBI nr database. The outermost circle represents the reference plasmid pPA166-2-MDR. The MDR region is highlighted with dotted lines. The acquired antimicrobial resistance genes, namely, conserved Inc_pRBL16_ backbone markers repA_IncpRBL16_ together with its iterons, *parB2*-*parA*, *che*, *pil*, and *ter* are annotated. (B) Alignment of the 78,304 bp MDR region in plasmid pPA166-2-MDR with the similar region in plasmid pR31014-IMP. Red, cyan, and orange arrows denote antimicrobial resistance genes, mobile elements, and other protein-encoding genes, respectively. Alignment of genetic contexts of *tmexCD3*-*toprJ3* (C) and *tet*(X6) (D) in plasmid pPA166-2-MDR with similar sequences. Red, cyan, and orange arrows denote antimicrobial resistance genes, mobile elements, and other protein-encoding genes, respectively. The Δ symbol indicates that the gene is truncated.

To decipher the genomic characterization, the genome of PA166-2 was extracted from overnight cultures by using the PureLink Genomic DNA Minikit (Invitrogen, Carlsbad, CA, USA) and sequenced by using the Illumina NextSeq 500 sequencing (2 × 150 bp) platform and the Nanopore MinION sequencer platform (14). The hybrid assembly of both sequencing reads was constructed using Unicycler v0.4.9b (15). The assembled genome of PA166-2 was annotated with the rapid antimicrobial susceptibility testing (RAST) tool and edited manually (16). Multilocus sequence typing was conducted by using the mlst software package (17). Antimicrobial resistance genes were analyzed by using ResFinder 2.1 (18). The genome of strain PA166-2 contained a 431,461 bp plasmid that was designated pPA166-2-MDR and a chromosome which was assembled into two contigs with lengths of 6,438,660 bp and 116,925 bp, respectively. The overall chromosome content of strain PA166-2 was comprised of 6,732 predicted open reading frames (ORFs), with a guanine-cytosine (GC) content of 65.6%. Antimicrobial resistance genes, including *fosA*, *catB7*, *bla*_OXA-50_, *aph(3′)-IIb*, and *bla*_PAO_, were detected on the chromosome of PA166-2. MLST analysis suggested that strain PA166-2 belonged to ST313. Plasmid pPA166-2-MDR contained 493 ORFs with a GC content of 56.3%. Two plasmids, pBM413 (CP016215) and pR31014-IMP (MF344571), both of which have similar backbones to that of pPA166-2-MDR, were retrieved from the NCBI nr database via a nucleotide Basic Local Alignment Search Tool (BLASTn) analysis (Fig. 1A). Plasmids belonging to the incompatibility group Inc_pRBL16_ that contained diverse collections of resistance genes were recently reported in Pseudomonas spp. (19). Conserved Inc_pRBL16_ backbone marker repA_IncpRBL16_ together with its iterons, *parB2*-*parA*, *che*, *pil*, and *ter*, were detected on pPA166-2-MDR, pBM413, and pR31014-IMP, suggesting that they all belonged to the Inc_pRBL16_ plasmid. An array of different resistance genes containing *tmexCD3*-*toprJ3*, *bla*_IMP-45_, *bla*_OXA-1_, *tet*(X6), *tet*(C), *mph*(E), *msr*(E), *armA*, *sul1* (2 copies), *catB3*, *qnrVC1*, *arr-3*, *floR*, *strAB* (2 copies), *ant(3′')-Ih-aac(6′)-IId*, *dfrA22e*, *aph(3′)-VIa*, *aph(4)-Ia*, *aac(3)-IVa*, and *aph(3′)-Ic* were found in plasmid pPA166-2-MDR. Notably, this is the first known report of the cooccurrence of *bla*_IMP-45_, *tet*(X6), and *tmexCD3*-*toprJ3* in one plasmid. The multidrug resistance (MDR) region containing all of these acquired resistance genes was 78,304 bp in length and was similar to the corresponding region in pR31014-IMP, except for the presence of a *ca.* 19 Kb region harboring *tet*(X6) in pPA166-2-MDR. As in other Inc_pRBL16_ plasmids, diverse mobile genetic elements, including Tn*As1*, *intI1* (2 copies), IS*CR1*, IS*Ec28*, IS*1349*, IS*Ec29*, IS*6100* (2 copies), IS*Ec59*, Tn*5393*, IS*Vsa3*, IS*Cfr1*, and IS*26* (4 copies) were detected in this MDR region (Figure 1B), suggesting that it was acquired via horizontal gene transfer and that active genetic recombination could have occurred in this region. A conjugation assay was performed via the filter mating method, using E. coli EC600 and fosfomycin-resistant P. aeruginosa PAO1 as the recipients. Transconjugants were selected on LB agar plates containing 1 mg/L meropenem and 600 mg/L rifampicin or 150 mg/L fosfomycin, respectively. Plasmid pPA166-2-MDR could not be transferred to P. aeruginosa and E. coli via direct conjugation under laboratory conditions.

*tet*(X6), which is a variant of the *tet*(X) gene that confers high-level tigecycline resistance, was first reported on an SXT/R391 element, ICE*Pgs6Chn1*, in Proteus sp. (20). Previous studies have demonstrated that *tet*(X6) is frequently associated with the genetic context of *tet*(X6)-*abh*-*guaA*-IS*Vsa3*, which is highly similar to that of other reported plasmid-borne *tet*(X) variants that are flanked by one or two IS*Vsa3* elements (20, 21). Likewise, pPA166-2-MDR carried *tet*(X6)-*abh*-*guaA*-IS*Vsa3* genetic content, and the IS*Vsa3* element upstream of *tet*(X6) was absent. Highly similar to that in ICE*Pgs6Chn1*, the *tet*(X6) in pPA166-2-MDR was downstream of a truncated Tn*5393* (Figure 1D). A BLASTn search in NCBI suggested that the *tet*(X) genes were absent on the Inc_pRBL16_ plasmids in the database. *tmexCD3*-*toprJ3* was also first reported in Proteus sp. on an SXT/R391 element, ICE*Pmi*ChnRGF134-1 (7). Previous studies have shown that most transposition units containing the *tmexCD3*-*toprJ3*-like gene clusters inserted into a similar site in the *umuC* gene (7, 22). In line with these findings, *tmexCD3*-*toprJ3* in plasmid pPA166-2-MDR was found to be inserted in *umuC*, constituting the genetic context of IS*Cfr1*-*tnfxB3*-*tmexC3*-*tmexD3*-*toprJ3*-△*umuC*. A BLASTn search with this genetic element in the NCBI nr database returned 8 hits (MF344570, KY883660, CP016215, CP086014, MF344568, CP073081, MN208062, and MF344571) with >98.5% identity at 100% coverage. All 8 of the sequences were from plasmids that belonged to the incompatibility group Inc_pRBL16_ (Figure 1C). The *bla*_IMP-45_ gene in pPA166-2-MDR was located directly downstream of the transposable element Tn*As1* and in a class 1 integron, In*786*, with the gene arrangement *intI1*-*aacA4*-*bla*_IMP-45_-*bla*_OXA-1_-*catB3*. In*786* was located within a Tn*6485b* transposon in pPA166-2-MDR. Similar genetic contexts were detected or reported in several other Inc_pRBL16_ plasmids, including pBM413 and pR31014-IMP (Figure 1B) (19, 23, 24). Our findings suggested that Inc_pRBL16_ was an important vector for the dissemination of last-line antibiotic resistance genes in Pseudomonas sp. The spread of plasmids like pPA166-2-MDR is of great concern for public health.

P. aeruginosa strains belonging to ST313 were widely disseminated across different continents (25). They have been described as intestinal colonizers in healthy individuals but were rarely reported from the poultry farm environment (26). The detection of such a strain in a chicken in this study suggested that this poultry could have been contaminated by human activities. Infections caused by P. aeruginosa are challenging to treat due to the intrinsic resistance of this bacterium to many antipseudomonal agents as well as its ability to acquire resistance determinants (2). ST313 P. aeruginosa were frequently reported to be associated with antimicrobial resistance genes, such as the carbapenemase genes *bla*_VIM_ and *bla*_KPC_ (27). However, the presence of Inc_pRBL16_ MDR plasmids in ST313 P. aeruginosa was not reported previously. The acquisition of the Inc_pRBL16_ plasmid carrying last-resort antimicrobial resistance genes *bla*_IMP-45_, *tet*(X6), and *tmexCD3*-*toprJ3* by P. aeruginosa pose considerable threats to public health.

In conclusion, this study reported, for the first time, the occurrence of last-resort antibiotic resistance determinants *bla*_IMP-45_, *tet*(X6), and *tmexCD3*-*toprJ3* on a single plasmid in P. aeruginosa from a chicken. The results of this study highlighted that Inc_pRBL16_ plasmids may serve as a reservoir for the dissemination of resistance genes. Control measures, such as strict supervision, the application of laws to control antibiotic use, and timely screening, should be implemented to prevent the further dissemination of such strains.

### Data availability.

The complete genome sequence of strain PA166-2 has been deposited in the NCBI GenBank database under the BioProject accession number PRJNA798590.

## References

[1] Azam MW, Khan AU. 2019. Updates on the pathogenicity status of Pseudomonas aeruginosa. Drug Discov Today 24:350–359. doi:10.1016/j.drudis.2018.07.003. 30036575

[2] Pang Z, Raudonis R, Glick BR, Lin T-J, Cheng Z. 2019. Antibiotic resistance in Pseudomonas aeruginosa: mechanisms and alternative therapeutic strategies. Biotechnol Adv 37:177–192. doi:10.1016/j.biotechadv.2018.11.013. 30500353

[3] Botelho J, Grosso F, Peixe L. 2019. Antibiotic resistance in Pseudomonas aeruginosa–mechanisms, epidemiology and evolution. Drug Resist Updat 44:100640. doi:10.1016/j.drup.2019.07.002. 31492517

[4] Lv L, Wan M, Wang C, Gao X, Yang Q, Partridge SR, Wang Y, Zong Z, Doi Y, Shen J, Jia P, Song Q, Zhang Q, Yang J, Huang X, Wang M, Liu J-H. 2020. Emergence of a plasmid-encoded resistance-nodulation-division efflux pump conferring resistance to multiple drugs, including tigecycline, in Klebsiella pneumoniae. mBio 11:e02930-19. doi:10.1128/mBio.02930-19. 32127452 PMC7064769

[5] Sun S, Gao H, Liu Y, Jin L, Wang R, Wang X, Wang Q, Yin Y, Zhang Y, Wang H. 2020. Co-existence of a novel plasmid-mediated efflux pump with colistin resistance gene mcr in one plasmid confers transferable multidrug resistance in Klebsiella pneumoniae. Emerg Microbes Infect 9:1102–1113. doi:10.1080/22221751.2020.1768805. 32401163 PMC8284978

[6] Wang C-Z, Gao X, Yang Q-W, Lv L-C, Wan M, Yang J, Cai Z-P, Liu J-H. 2021. A novel transferable resistance-nodulation-division pump gene cluster, tmexCD2-toprJ2, confers tigecycline resistance in Raoultella ornithinolytica. Antimicrob Agents Chemother 65:e02229-20. doi:10.1128/AAC.02229-20. 33495220 PMC8097428

[7] Wang Q, Peng K, Liu Y, Xiao X, Wang Z, Li R. 2021. Characterization of TMexCD3-TOprJ3, an RND-type efflux system conferring resistance to tigecycline in Proteus mirabilis, and its associated integrative conjugative element. Antimicrob Agents Chemother 65:e02712-20. doi:10.1128/AAC.02712-20. 33875423 PMC8218640

[8] Sun S, Wang Q, Jin L, Guo Y, Yin Y, Wang R, Bi L, Zhang R, Han Y, Wang H. 2022. Identification of multiple transfer units and novel subtypes of tmexCD-toprJ gene clusters in clinical carbapenem-resistant Enterobacter cloacae and Klebsiella oxytoca. J Antimicrob Chemother 77:625–632. 34893837 10.1093/jac/dkab434

[9] Hirabayashi A, Dao TD, Takemura T, Hasebe F, Trang LT, Thanh NH, Tran HH, Shibayama K, Kasuga I, Suzuki M. 2021. A transferable IncC-IncX3 hybrid plasmid cocarrying bla NDM-4, tet (X), and tmexCD3-toprJ3 confers resistance to carbapenem and tigecycline. Msphere 6:e00592-21. doi:10.1128/mSphere.00592-21. 34346701 PMC8386453

[10] Qin S, Peng J, Deng R, Peng K, Yan T, Chen F, Li R. 2021. Identification of two plasmids coharboring carbapenemase genes and tmexCD1-toprJ1 in clinical Klebsiella pneumoniae ST2667. Antimicrob Agents Chemother 65:e00625-21. doi:10.1128/AAC.00625-21. 33846130 PMC8316132

[11] Li R, Peng K, Xiao X, Liu Y, Peng D, Wang Z. 2021. Emergence of a multidrug resistance efflux pump with carbapenem resistance gene bla VIM-2 in a Pseudomonas putida megaplasmid of migratory bird origin. J Antimicrob Chemother 76:1455–1458. doi:10.1093/jac/dkab044. 33758948

[12] Sun J, Chen C, Cui C-Y, Zhang Y, Liu X, Cui Z-H, Ma X-Y, Feng Y, Fang L-X, Lian X-L, Zhang R-M, Tang Y-Z, Zhang K-X, Liu H-M, Zhuang Z-H, Zhou S-D, Lv J-N, Du H, Huang B, Yu F-Y, Mathema B, Kreiswirth BN, Liao X-P, Chen L, Liu Y-H. 2019. Plasmid-encoded tet (X) genes that confer high-level tigecycline resistance in *Escherichia coli*. Nat Microbiol 4:1457–1464. doi:10.1038/s41564-019-0496-4. 31235960 PMC6707864

[13] He T, Wang R, Liu D, Walsh TR, Zhang R, Lv Y, Ke Y, Ji Q, Wei R, Liu Z, Shen Y, Wang G, Sun L, Lei L, Lv Z, Li Y, Pang M, Wang L, Sun Q, Fu Y, Song H, Hao Y, Shen Z, Wang S, Chen G, Wu C, Shen J, Wang Y. 2019. Emergence of plasmid-mediated high-level tigecycline resistance genes in animals and humans. Nat Microbiol 4:1450–1456. doi:10.1038/s41564-019-0445-2. 31133751

[14] Li R, Xie M, Dong N, Lin D, Yang X, Wong MHY, Chan EW-C, Chen S. 2018. Efficient generation of complete sequences of MDR-encoding plasmids by rapid assembly of MinION barcoding sequencing data. Gigascience 7:1–9. doi:10.1093/gigascience/gix132. PMC5848804 29325009

[15] Wick RR, Judd LM, Gorrie CL, Holt KE. 2017. Unicycler: resolving bacterial genome assemblies from short and long sequencing reads. PLoS Comput Biol 13:e1005595. doi:10.1371/journal.pcbi.1005595. 28594827 PMC5481147

[16] Overbeek R, Olson R, Pusch GD, Olsen GJ, Davis JJ, Disz T, Edwards RA, Gerdes S, Parrello B, Shukla M, Vonstein V, Wattam AR, Xia F, Stevens R. 2014. The SEED and the rapid annotation of microbial genomes using subsystems technology (RAST). Nucleic Acids Res 42:D206–D214. doi:10.1093/nar/gkt1226. 24293654 PMC3965101

[17] Seemann T. MLST. https://github.com/tseemann/mlst. Accessed 1 September 2021.

[18] Kleinheinz KA, Joensen KG, Larsen MV. 2014. Applying the ResFinder and VirulenceFinder web-services for easy identification of acquired antibiotic resistance and E. coli virulence genes in bacteriophage and prophage nucleotide sequences. Bacteriophage 4:e27943. doi:10.4161/bact.27943. 24575358 PMC3926868

[19] Jiang X, Yin Z, Yuan M, Cheng Q, Hu L, Xu Y, Yang W, Yang H, Zhao Y, Zhao X, Gao B, Dai E, Song Y, Zhou D. 2020. Plasmids of novel incompatibility group IncpRBL16 from Pseudomonas species. J Antimicrob Chemother 75:2093–2100. doi:10.1093/jac/dkaa143. 32395746

[20] He D, Wang L, Zhao S, Liu L, Liu J, Hu G, Pan Y. 2020. A novel tigecycline resistance gene, tet (X6), on an SXT/R391 integrative and conjugative element in a Proteus genomospecies 6 isolate of retail meat origin. J Antimicrob Chemother 75:1159–1164. doi:10.1093/jac/dkaa012. 32016288

[21] Cheng Y, Chen Y, Liu Y, Song J, Chen Y, Shan T, Xiao Y, Zhou K. 2021. Detection of a new tet (X6)-encoding plasmid in Acinetobacter towneri. J Glob Antimicrob Resist 25:132–136. doi:10.1016/j.jgar.2021.03.004. 33762210

[22] Wang C-Z, Gao X, Lv L-C, Cai Z-P, Yang J, Liu J-H. 2021. Novel tigecycline resistance gene cluster tnfxB3-tmexCD3-toprJ1b in Proteus spp. and Pseudomonas aeruginosa, co-existing with tet (X6) on an SXT/R391 integrative and conjugative element. J Antimicrob Chemother 76:3159–3167. doi:10.1093/jac/dkab325. 34508611

[23] Liu J, Yang L, Chen D, Peters BM, Li L, Li B, Xu Z, Shirtliff ME. 2018. Complete sequence of pBM413, a novel multidrug resistance megaplasmid carrying qnrVC6 and blaIMP-45 from Pseudomonas aeruginosa. Int J Antimicrob Agents 51:145–150. doi:10.1016/j.ijantimicag.2017.09.008. 28923459

[24] Wang Y, Wang X, Schwarz S, Zhang R, Lei L, Liu X, Lin D, Shen J. 2014. IMP-45-producing multidrug-resistant Pseudomonas aeruginosa of canine origin. J Antimicrob Chemother 69:2579–2581. doi:10.1093/jac/dku133. 24777897

[25] Rada AM, De La Cadena E, Agudelo CA, Pallares C, Restrepo E, Correa A, Villegas MV, Capataz C. 2021. Genetic diversity of multidrug-resistant Pseudomonas aeruginosa isolates carrying blaVIM–2 and blaKPC–2 genes that spread on different genetic environment in Colombia. Front Microbiol 12:663020. doi:10.3389/fmicb.2021.663020. 34512563 PMC8432936

[26] Valenza G, Tuschak C, Nickel S, Krupa E, Lehner-Reindl V, Höller C. 2015. Prevalence, antimicrobial susceptibility, and genetic diversity of Pseudomonas aeruginosa as intestinal colonizer in the community. Infect Dis (Lond) 47:654–657. doi:10.3109/23744235.2015.1031171. 25832457

[27] Libisch B, Watine J, Balogh B, Gacs M, Muzslay M, Szabó G, Füzi M. 2008. Molecular typing indicates an important role for two international clonal complexes in dissemination of VIM-producing Pseudomonas aeruginosa clinical isolates in Hungary. Res Microbiol 159:162–168. doi:10.1016/j.resmic.2007.12.008. 18280707

